# Cervical Tissue Hydration Level Monitoring by a Resonant Microwave Coaxial Probe

**DOI:** 10.3390/s22239527

**Published:** 2022-12-06

**Authors:** Heungjae Choi, Emilia Barker, Ali A. Abduljabar, Dilly Anumba, Adrian Porch

**Affiliations:** 1 School of Engineering, Cardiff University, 14-17 The Parade, Cardiff CF24 3AA, UK; 2 School of Clinical Dentistry, University of Sheffield, 19 Claremont Crescent, Sheffield S10 2TA, UK; 3 College of Engineering, University of Basrah, Basrah 61002, Iraq; 4 Department of Oncology and Metabolism, JW4/40, Level 4, Jessop Wing, Tree Root Walk, Sheffield S10 2SF, UK

**Keywords:** harmonic resonance, microwave, preterm birth, sensing, tissue hydration

## Abstract

Cervical tissue hydration level is one of the most important parameters to monitor in the early diagnosis of preterm birth. Electrical-impedance-spectroscopy-based techniques are often used, but they suffer from limited accuracy. Open microwave coaxial probes have been widely used as a broadband dielectric characterization technique for human tissue samples due to their versatility, but with limited accuracy due to their nonresonant nature. In this work, a resonant microwave open coaxial probe with multiple harmonic resonances is proposed as a sensing platform for tissue-hydration-level monitoring. The mechanical design was analyzed and verified by finite-element full 3D electromagnetic simulation and experiments. Dominant sources of errors and the ways to mitigate them were discussed. In vitro experiments were carried out on human cervix samples to verify the precision and accuracy by comparing the results to a commercial skin-hydration sensor. The proposed sensor shows mean fractional frequency shift of (3.3 ± 0.3) × 10^−4^ per unit % over the entire data. This translates into an absolute frequency shift ($\Delta f_{N})$ of 252 ± 23 kHz/%, 455 ± 41 kHz/%, and 647 ± 57 kHz/% at second, fourth, and sixth harmonic resonance, respectively.

## 1. Introduction

Every year, an estimated 15 million babies are born preterm, defined as 37 completed weeks of gestation [1]; this is more than 1 in 10 babies and is rapidly rising. Spontaneous preterm birth and the relevant complications were responsible for about 1 million deaths in 2015, becoming the leading cause of death among children under 5 years of age [2]. Unfortunately, the majority of survivors end up experiencing life-long developmental delay with breathing, vision, and hearing problems [3].

Microstructural changes to the cervix such as cervical softening, shortening, and dilation, are known to be common indicators of preterm birth [4]. Tissue hydration, collagen structure, and tissue elasticity progressively change with cervical microstructure changes as pregnancy progresses [5,6].

Tissue hydration can be measured and monitored using several direct and indirect methods: (1) dilution techniques based on laboratory analysis of a tracer concentration in blood and urine samples [7], (2) biological impedance and conductance methods, including single-frequency bioelectrical impedance analysis [8] and biological impedance spectroscopy, which measures resistance and reactance over wide range of frequencies [9,10], and (3) total-body electrical conductivity using a solenoid that generates a time-varying electromagnetic field and eddy currents [11]. There were some attempts in development of segmental bioelectrical impedance methods; however, their accuracy was not adequate [12]. Medical imaging techniques, such as magnetic resonance imaging (MRI) and ultrasonography, are potentially sensitive to water content in the tissue and are widely used for visualization of internal structures and finding lesions, but not for assessment of water content [13,14]. However, despite attempts to use water-selective modes in contrast MRI [15,16], in general, the imaging techniques are not suitable for quantitative evaluation of water content. Nuclear magnetic resonance analysis of microwave-dried meat samples [17] was used for fast determination of fat and water content, but it is not suitable for in vivo experiments. The same applies to optical infrared reflectance spectroscopy [18]. Optical digital imaging is easy and straightforward, but imprecise and semi-quantitative [19]. Electrical impedance spectroscopy has shown little clinical utility [20], and acoustic attenuation measurement requires tissue homogeneity and shows wide intra-subject variability [21]. Stromal differentiation using Raman spectroscopy is expensive and semi-quantitative [22].

Magnetic induction spectroscopy is another emerging technique that shows promising results in cervical tissue measurements [23,24,25]. Some commercial instruments are able to measure the water content of the skin based on conductance measurements, such as Skicon (I.B.S. Co., Ltd., Hamamatsu, Japan), or capacitance, such as Corneometer^®^ (Courage Khazaka electronic GmbH, Cologne, Germany) and NOVA Dermal Phase Meter (Nova Technology Corporation, Broussard, LA, USA) [26]. Such corneometers are claimed to have ±3% accuracy over the measurement frequency of 0.9–1.2 MHz [27].

Microwave dielectric spectroscopy is a useful and powerful technique in the characterization, sensing, and monitoring of human tissue properties due to its key advantages such as nondestructive, noninvasive, and label-free measurements, as well as rapid and focused power delivery capability for therapeutic applications [28,29,30,31,32,33,34,35,36,37]. The high dielectric constant of water produces high dielectric contrast when combined with other materials, such as human tissues, making dielectric spectroscopy a strong candidate for cervix tissue hydration monitoring.

The commonly used microwave cavity perturbation technique would provide high accuracy at a selected frequency among available dielectric characterization methods, but it requires a bulky resonator and specific sample shape and volume; therefore, it is not suitable for in vivo sensing and monitoring [38,39]. Although less accurate due to its nonresonant nature, the coaxial reflectance probe is best suited for lossy samples such as liquids and malleable samples due to its contact-based sensing mechanism and broadband characteristics. Therefore, it has been a popular choice for several decades in biological tissue characterization [40,41,42,43,44,45,46,47,48,49,50,51]. A novel coupling technique allowed transmission measurements from one end of a half-wavelength coaxial resonator, which improved the dynamic range while allowing the evanescent field at the sample end of the resonator [52,53,54,55,56,57,58]. A combination of high accuracy from the resonator-based perturbation mechanism, convenience of open coaxial probe and its form factor, and broadband information obtained from harmonic resonances will constitute an ideal technique for noninvasive in vivo cervix tissue hydration monitoring.

Design, mechanical construction, and characterization via simulation and measurement of the two-port coaxial harmonic resonance probe and relevant factors that affect accuracy are discussed in Section 2 and Section 3, including the details of sample preparation, test procedure, and data processing routine. The results comparing the proposed technique and the commercial Corneometer^®^ are summarized and discussed in Section 4, followed by conclusions in Section 5.

## 2. Probe Design and Characterization

### 2.1. Advantages of Two-Port Transmission Measurement

The complex permittivity of the samples under test are measured by their perturbation of the electric field at the open end of the coaxial resonator. There are several advantages of choosing the transmission (two-port) over the reflection (one-port) technique for resonator measurements of material properties. The main advantage is that it is possible to use weak coupling, which allows much simpler conversion of loaded quality factor to unloaded quality factor without the need for careful calibration. A weak coupling also means that the coupling structure does not perturb the electromagnetic field within the resonator, therefore minimizing the impact of the coupling structures in the material properties extraction. Another huge advantage is that, similar to a resonant cavity, in the two-port measurement technique, the dielectric properties of the sample can be extracted by simple ‘relative’ measurement between *air* and *sample*. This allows us to avoid solving a complicated inverse problem (that is, calculating dielectric properties from the measured admittance) and reduce the requirement of the vector network analyzer and calibration procedure [59,60,61]. In other words, we can simply utilize a low-cost scalar network analyzer in extracting complex permittivity, so it is possible to design a miniaturized portable diagnostic system around the resonator.

### 2.2. Two-Port Harmonic Resonance Coaxial Probe Design

**Figure 1:**
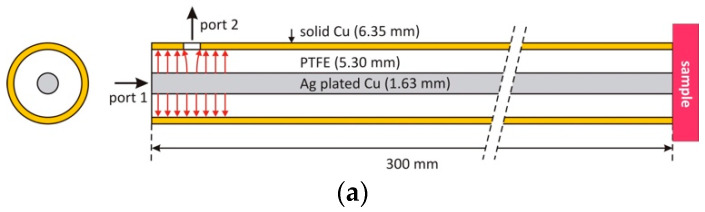

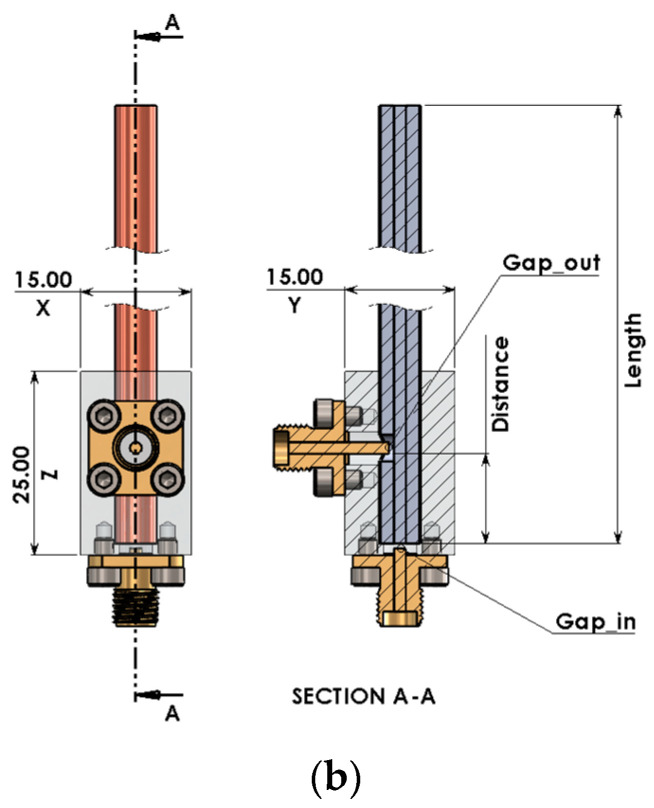
Mechanical construction of the two-port coaxial harmonic resonance probe. (**a**) Coaxial resonator formed from an open-ended coaxial cable (RG401, with material diameters shown), and (**b**) 3D CAD model of the complete coaxial resonant probe with break view in the length of coaxial cable. Cross-section A-A shows the detailed coupling structure inside the aluminum fixture that hosts two SMA connectors and the RG401 cable (all units in mm).

To avoid any non-TEM modes and to ensure broadband operation, the cross-sectional dimensions of the probe should be much smaller than the wavelength. As shown in Figure 1, PTFE-filled RG401 (inner conductor diameter 1.63 ± 0.03 mm, outer conductor 6.35 ± 0.03 mm) 50 Ω coaxial cable is chosen in the design for several reasons, but mainly because of its wide availability, low cost, and rigidity for cervix tissue hydration monitoring application. Both ends of the coaxial cable are cut and flattened to form a half-wavelength resonator, where one end will be in touch with the sample under test while the opposite end will have input and output coupling ports. A small hole (e.g., one with a radius of 2 mm) is required at the outer conductor and the PTFE of the coaxial cable at port 2′s location in Figure 1a to allow coupling. The relationship between the length and resonant frequencies can be defined as follows [39],

(1) $$f_{N}=\frac{N}{2l}\cdot \frac{c}{\sqrt{\varepsilon_{r}}}\;,$$

where $f_{N}$ is the harmonic resonant frequency, *l* is the length of the coaxial resonator, *N* is the harmonic number (defined to be the number of half wavelengths along the length), *c* is the speed of light in vacuo, and $\varepsilon_{r}$ is the relative permittivity of the dielectric filler material (PTFE). According to (1), a length of 300 mm gives a fundamental resonant frequency of about 350 MHz, with higher-order harmonics at integer multiples of the fundamental frequency. This length allows the investigation of the dielectric properties of the target sample under test (i.e., water-based tissues) over RF (low *N*) and the lower microwave frequency range (high *N*).

Capacitive coupling with the electric field (E-field), as shown schematically with the red arrows in Figure 1a, is made at one end of the cable using a short, extruded inner conductor of an SMA connector, and the sample is placed at the other end. As illustrated in Figure 1b, a rectangular aluminum fixture (15 ×15 × 25 mm^3^) is used to hold the coaxial cable and two coupling ports of the SMA connectors. Since the coaxial cable is open circuit at each end, the E-field in each resonant mode is maximum at both ends, yielding effective microwave coupling that increases with increasing frequency, and effective E-field coupling with the sample for assessment of its dielectric properties. To achieve weak coupling, as discussed in the previous section, the gap between the inner conductor of the coaxial resonator and each coupling port (*Gap_in* and *Gap_out*) was adjusted so that both port 1 and 2 have symmetric coupling strength and produce insertion loss (S_21_) of 30 dB at the fundamental resonant frequency. This requires *Gap_in* and *Gap_out* to be about 1 mm. The distance between the two ports (*Distance*) is chosen to be 13 mm, so that there is little direct coupling between two ports. Due to this unique coupling structure, one of the harmonic resonances (e.g., *N* = 10) is diminished when the distance equates to the quarter wavelength of that specific harmonic frequency. This will be further investigated with the aid of 3D finite element simulation in the next section.

### 2.3. Simulation and Characterization

**Figure 2:**
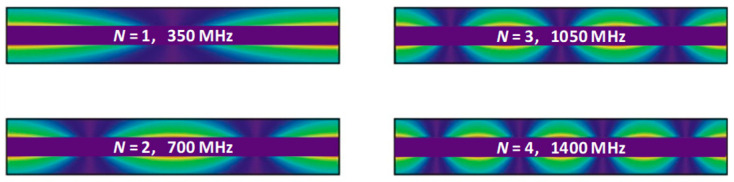
Calculated E-field distribution of the first four modes within an open-ended coaxial resonator (Scale: red is high, purple is low).

The E-field magnitude along the length and inside of the coaxial resonant probe for the first four modes (*N* is the mode number) calculated and illustrated in Figure 2 based on the theoretical expression for electric field, i.e., simple sinusoids of degreasing wavelength (scale: red is high, purple is low). Both ends of the coaxial cable are assumed to be an open circuit; hence, Figure 2 shows maximum E-field at these ends.

**Figure 3:**
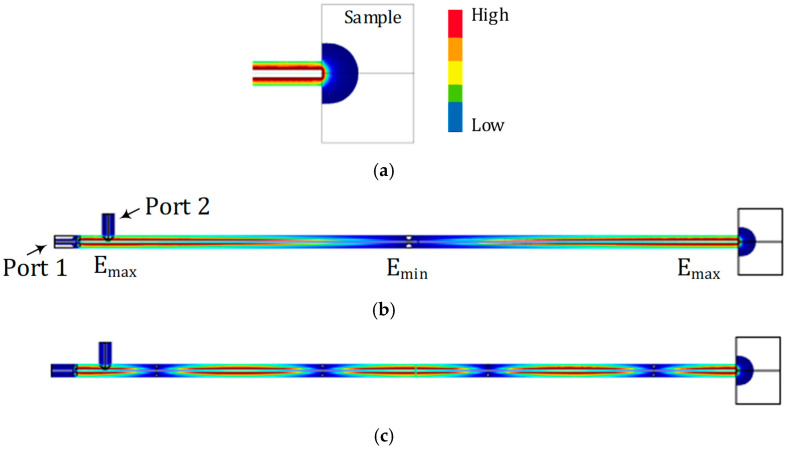
Full 3D simulation results modeled by using COMSOL Multiphysics. E-field distribution within the resonator and fringing field inside the sample is plotted (**a**) at 350 MHz (*N* = 1) zoomed in to show the probe-sample boundary, (**b**) at 350 MHz (*N* = 1), and (**c**) at 1400 MHz (*N* = 4).

The probe in full 3D geometry is also simulated in COMSOL Multiphysics by using the model illustrated in Figure 1 and the E-field distribution is plotted at 350 MHz (*N* = 1) in Figure 3a,b and at 1400 MHz (*N* = 4) in Figure 3c, respectively. The model is a one-to-one replica of the fabricated probe, including input and output ports for frequency domain analysis, except that the aluminum fixture is not considered, as it does not affect the results. The sample on the right-hand side of the probe is set to a cylinder of air ($\varepsilon_{r}=1)$, and $\varepsilon_{r}=2$ is used for PTFE. The outer conductor of the coaxial probe is set to have a Perfect Electric Conductor (PEC) boundary. The E-field scale is adjusted (somewhat exaggerated) for better illustration, with red indicating a high E-field and blue indicating a low E-field. As shown in Figure 3a, the sample under test interacts with the evanescent E-field at the proximity of the open-ended coaxial probe tip. This volume of interaction determines the sample size and thickness. The sample needs to be thick enough so that all available fields in the vicinity of the probe are located inside the sample under test. Practically, material thickness of four times the aperture diameter is recommended. Figure 3b,c illustrate the electric field distribution within the coaxial probe for fundamental and fourth harmonic resonant frequencies, respectively.

**Figure 4:**
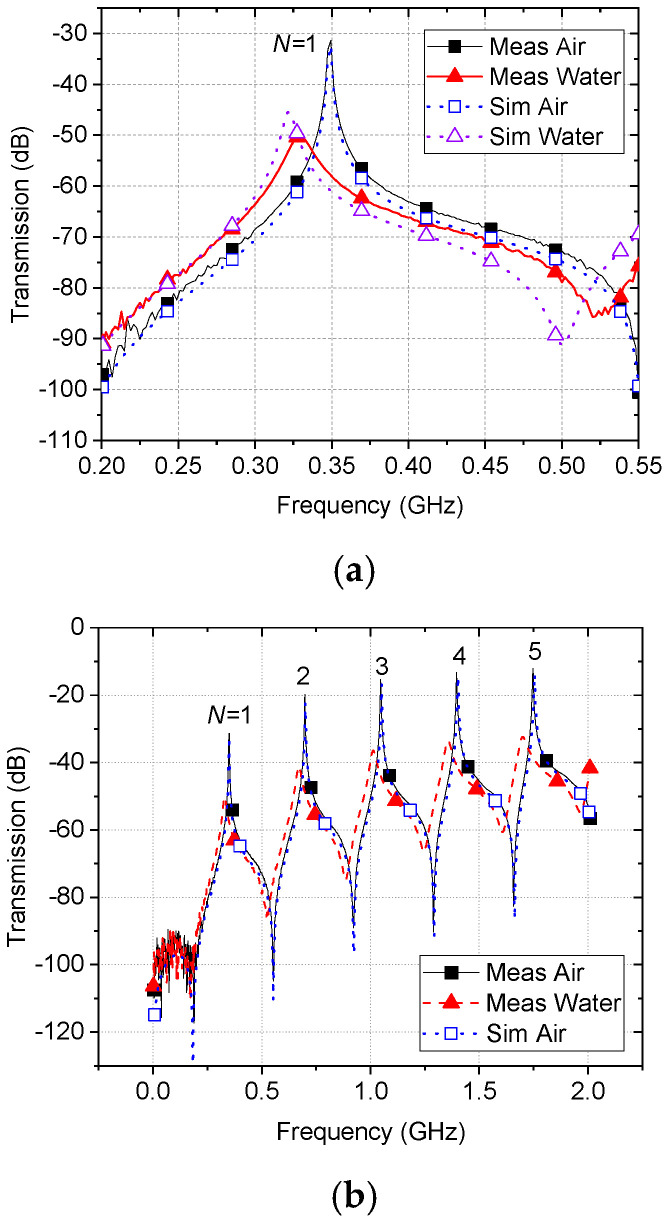

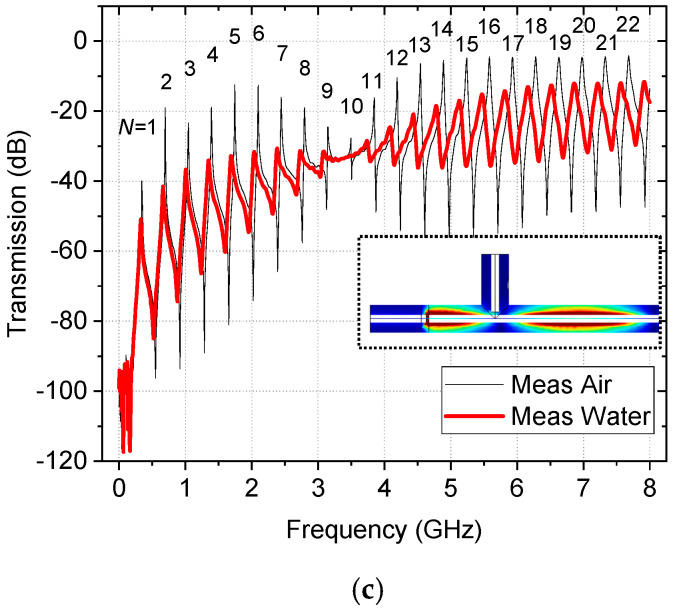
Simulation (air) and measurement (air and water) results, (**a**) at the fundamental resonant frequency of 350 MHz, (**b**) up to 2 GHz showing 5 harmonic resonances, and (**c**) broadband response up to 8 GHz including 22 harmonic resonances, measured with and without water. Inset is the field distribution at 3500 MHz (*N* = 10) zoomed in around the two coupling ports.

Simulated and measured broadband transmission characteristics with *air* and *water* as samples are shown in the frequency domain in Figure 4, illustrating the harmonic resonances at integer multiples of the fundamental frequency of 350 MHz. For water simulation, a specific water model of H_2_O (Water)[liquid, distilled, 2 to 50 GHz, tested at 25 °C (298 K)] from COMSOL Material Library was used. In Figure 4a, the blue dotted line with the empty square symbol is the COMSOL Multiphysics simulation for an air-terminated coaxial resonant probe. The black solid line with solid squares is the experimental result for the air-terminated probe, showing almost perfect agreement with the simulation, and the red dashed line with solid triangle is the experimental result when the end of the probe is fully immersed in de-ionized water. The purple dotted line with an empty triangle shows the simulated water response, where the discrepancy comes from the difference in material properties. All experimental data are taken using a vector network analyzer (Keysight Fieldfox N9923A). A bespoke LabVIEW program (National Instruments) is used to record and extract continuous changes in resonant frequency, 3 dB (half-power) bandwidth, and peak power at resonance for each resonant mode. With water as the sample under test, for each mode, the resonant frequency shifts downwards, the 3 dB bandwidth increases, and there is a decrease in the peak power level. We expect the amount of frequency shift and increase in 3 dB bandwidth to be proportional to the amount of water content in the sample. Figure 4b shows harmonic resonant modes up to the fifth harmonic resonance.

At the frequency where the distance between the two coupling ports equates to the quarter-wavelength, in this case at 3500 MHz, the E-field minimum will be aligned with port 2 so that this mode is suppressed. This is clearly visible from the inset field distribution in Figure 4c, which shows broadband characteristics, including 22 harmonic resonances up to 8 GHz. Since there are many modes with high-quality factor available and the probe is intended to be used for tissue hydration monitoring, this missing null is not critical in investigating the broadband dielectric properties of water-based tissue samples.

Table 1 summarizes the measured characteristics of the coaxial resonant probe when it is air-terminated, where each column of Frequency, f_0_, Bandwidth, and Loss indicates expected harmonic resonant frequencies, measured harmonic resonant frequencies, 3 dB bandwidth, and peak insertion loss, respectively. Q_L_ and Q_0_ indicate loaded and unloaded quality factors. The unloaded quality factor of each mode varies from a minimum of 351 to maximum of 754, allowing us to measure the change in tissue-hydration level with enough fidelity.

## 3. Experimental Section

### 3.1. Sample Collection and Preparation

**Figure 5:**
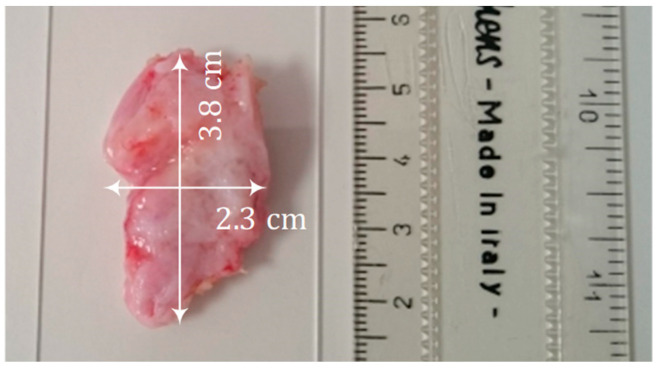
Cervix tissue sample with a ruler (left unit is in cm).

Human cervical samples were obtained following hysterectomies for benign gynecological conditions that do not affect the cervix at the Royal Hallamshire Hospital (Sheffield Teaching Hospitals, National Health Service Foundation Trust, UK). Patients gave informed written consent before the operation for use of a portion of the extirpated cervical tissue for research, as approved by the North Sheffield research ethics committee (Ref-08/H1310/35) admissions procedure. Cervical samples were stored in sterile PBS supplemented with Penicillin-Streptomycin and Fungizone in 4 °C. The area of the samples varied between 2 × 2 cm^2^ and 4 × 4 cm^2^. Figure 5 shows an example of the cervical tissue sample. The cervical samples were 5–6 days old when used for experiments. For reference, anonymized batch numbers of the four tested cervical samples were sample A (030215), sample B (DT6070), sample C (WE4388), and sample D (180318).

### 3.2. Experimental Setup and Test Procedure

**Figure 6:**
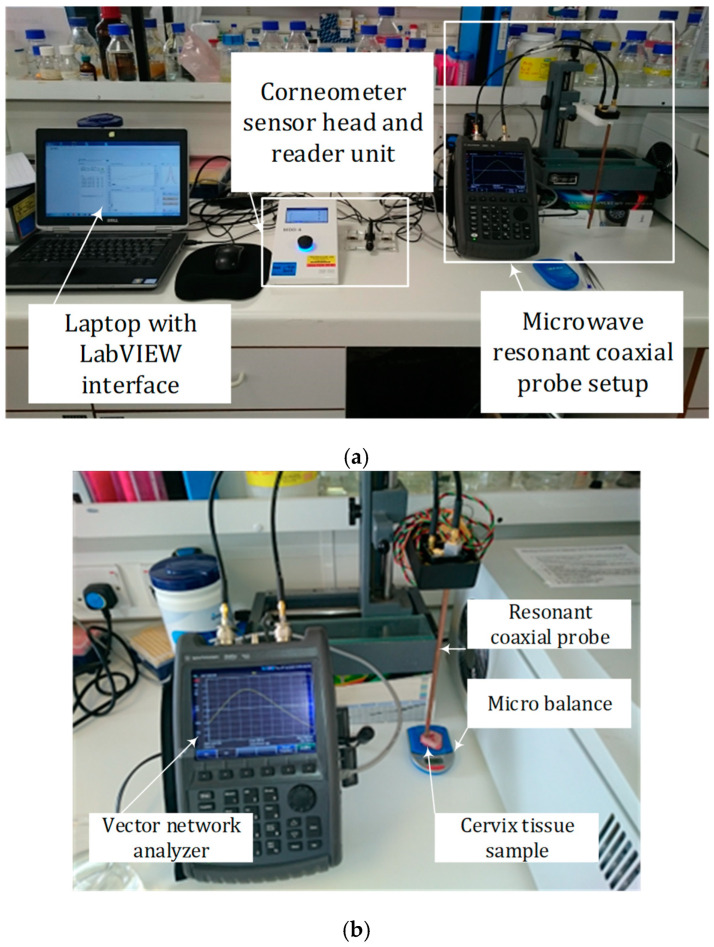
(**a**) Experimental setup, and (**b**) detailed resonant coaxial probe measurement setup including cervical tissue sample placed on a micro balance to monitor contact pressure.

The experimental setup consisted of the proposed resonant coaxial probe, a vector network analyzer (FieldFox, Keysight Technologies, Santa Rosa, CA, USA) that is controlled by LabVIEW user interface on a laptop computer, and a Corneometer as a reference hydration measurement. The setup is shown in Figure 6a. The outer conductor of the resonant coaxial probe (copper part) was coated with Parylene (Para Tech Coating Ltd., Northampton, UK) to avoid any contamination of samples. On the day of the experiment, each sample piece was weighed immediately after removal from the storage container using electronic lab micro balance. This initial weight was later referred to as 100% hydration, and the samples were left to undergo natural drying processes. Each sample was then weighed at every time point, before the microwave measurements were taken, and the hydration level was later calculated as fraction of its initial weight. Additionally, the Corneometer (model MDD4 with CM825 probe, Courage + Khazaka electronic GmbH, Köln, Germany) was used, and the moisture on the surface of the tissue was also recorded. Corneometer measurements were repeated 6 times at each time point to calculate average and standard deviation.

Each piece of cervical sample was placed on a micro balance (Pocket balance TEE, KERN & SOHN GmbH, Balingen, Germany) which acted as a force sensor, as shown in Figure 6b. The probe was lowered and pressed against the sample with force of 5.0 ± 0.5 g. Up to the 11^th^ harmonic resonances (*N* = 11, from 350 MHz to 3850 MHz) were collected, except for the diminished 10^th^ harmonic component for the reason explained previously. Fifteen measurements were taken at each harmonic resonance every 30 min for the duration of 3 to 48 h (when dry or the water contents dropped to below 40%) at room temperature.

### 3.3. Potential Confounders and Mitigation

There are two main types of errors. One is measurement-technique dependent, and the other is sample dependent. As we discussed in Section 2, one major advantage of the proposed technique is that it does not require calibration of the test instrument (e.g., the vector network analyzer) because it is a ‘relative’ measurement technique similar to the resonant cavity perturbation method. A few of the most significant sample-dependent errors include temperature and other atmospheric conditions, probe-sample pressure (including quality of probe-sample contact), and sample heterogeneity [28].

#### 3.3.1. Temperature and Humidity

It is well known that the dielectric properties are temperature dependent; therefore, the room temperature was carefully monitored during the experiment. The room temperature and the humidity were controlled within 21.3 ± 0.7 °C and 31.6 ± 1.1%, respectively, over the course of the experiment. Temperature dependence not only applies to the sample under test but also to the dielectric materials comprising the probe itself. In addition to this, the metallic components will also contract and expand according to the temperature, therefore affecting the resonance parameters. To characterize the temperature dependence of the fabricated resonant coaxial probe, the air-terminated probe (i.e., no sample) was placed inside an incubator (Memmert Cooled incubator, 5–70 °C, Schwabach, Germany) and a temperature ramp experiment was carried out over the range from 20 to 40 °C alongside continuous collection of the resonance parameters (f_0_, bandwidth, loss, Q). To minimize the opening in the hysteresis curve due to temperature lag during a series of temperature ramp experiments, the temperature varied from 20 °C to 40 °C and back to 20 °C over a period of 12 h, while continuously collecting the resonance parameters. For example, through linear regression analysis between temperature and resonant frequency, a temperature coefficient of *N* × (26.3 ± 1.1) kHz/°C was obtained, where *N* is the harmonic number. This was used to calibrate the temperature dependence of the probe.

#### 3.3.2. Quality of Probe-Sample Contact

**Figure 7:**
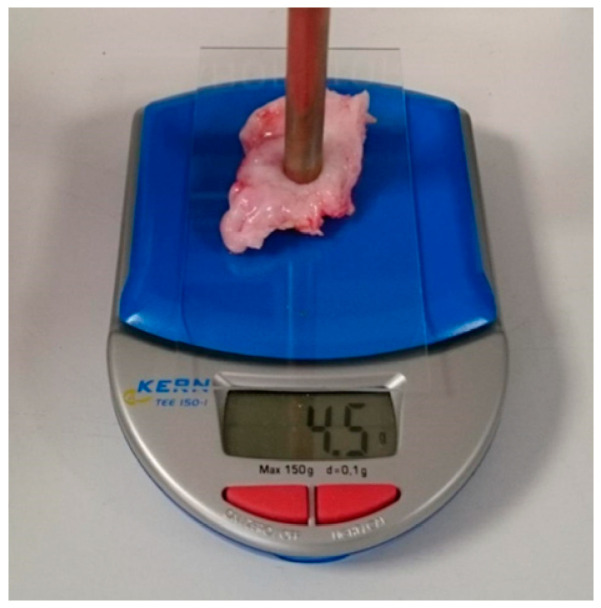
A photo showing cervix tissue-probe contact.

As shown in Figure 6, the resonant coaxial probe was placed vertically on a linear stage to ensure repeatable and consistent probe-sample contact pressure. The open end of the coaxial sensor was directly above the sample, and *z*-axis movement was used to move the probe up and down. A piece of sample on a glass slide was placed on a micro balance to monitor the probe-tissue contact pressure, as shown in Figure 7. The probe was moved down to make contact with a sample until the same force was applied in every contact, i.e., when the weight on display reached 5.0 ± 0.5 g, to minimize any airgap between the probe and the sample and achieve consistent quality of contact.

## 4. Data Analysis and Results

**Figure 8:**
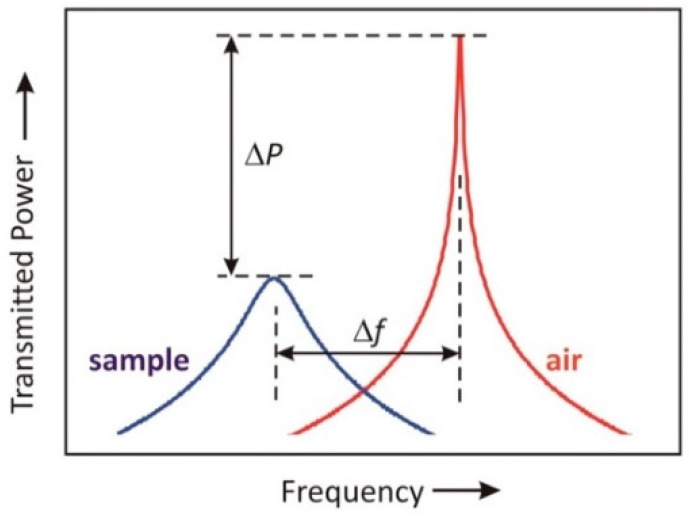
Microwave resonator perturbation diagram between air and sample measurement.

A basic concept of microwave perturbation in the assessment of hydration of the sample is summarized in Figure 8. The change in resonant frequency Δ*f* and power level Δ*P* are calculated, referenced to the air-terminated probe, for each resonant mode. Resonator perturbation theory tells us that the fractional frequency shift, for example, is

(2) $$\Delta f_{N}/f_{N,air}\approx A\left(\varepsilon_{\mathrm{eff}}-1\right),$$

where *A* is a dimensionless constant that depends weakly on the mode number *N* and $\varepsilon_{\mathrm{eff}}$ is the effective dielectric permittivity of the water-borne sample. In (2), a change in the amount of water in the tissue sample will affect $\varepsilon_{\mathrm{eff}}$, therefore affecting the resonant frequency.

Since the permittivity of water dominates over that of the host tissue material, if the volume fraction of water is defined to be *v*, then we may write that $\Delta f_{N}/f_{N,air}\;$≈ *B* + *Cv*, where *B* and *C* are dimensionless constants with *B* << *C*. This allows us to infer *v* from simple linear regression. Similar analysis may be performed on the change in power Δ*P* or the change in 3 dB bandwidth to determine *v*, but in practice, the resonant frequency shift yields the most reliable and precise data.

**Figure 9:**
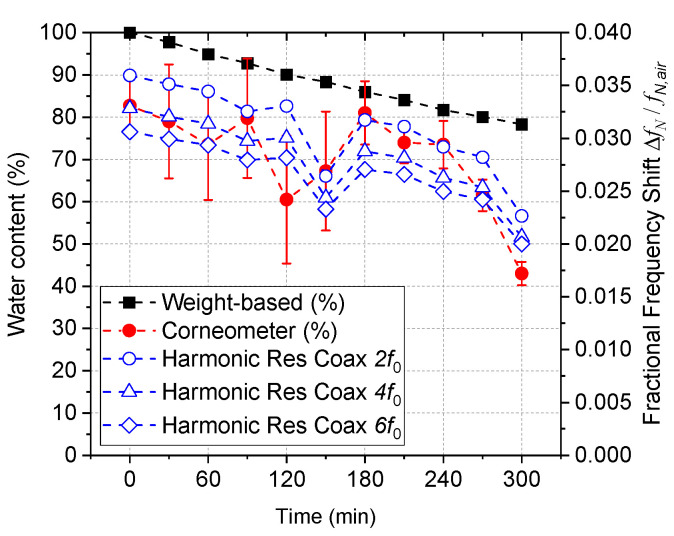
Water content over time of the cervix sample A (03215) measured by Corneometer (left *Y*-axis) and the harmonic resonance coaxial probe (right *Y*-axis) with relative weight-based water content (left *Y*-axis).

Figure 9 shows water content measurement of the cervical sample A (030215) over 6 h, during which the tissue dried naturally. Regarding the harmonic resonance coaxial probe, only data for the even harmonics at 700 MHz (second harmonic), 1400 MHz (fourth harmonic), and 2100 MHz (sixth harmonic) are shown in each plot, but measurements are taken routinely for the first nine modes, all showing similar trends. Fractional frequency shift is plotted, since this parameter reflects the effective dielectric constant $\varepsilon_{\mathrm{eff}}$ of the sample as in Equation (2), which has only a weak dependence on frequency at these low microwave frequencies, i.e., over the 1 to 2 GHz range, decreasing slightly with increasing mode number, as shown in Figure 9. As can be seen from the difference between the relative weight-based water content and the Corneometer results, the loss of water content was greater on the surface than the total water loss during the relative weight-based calculation method. This was expected because the cervical tissue does not have any protective layer, unlike skin. As the fringing electric field of the open coaxial probe has a penetration depth of only a few millimeters, like the Corneometer, the Corneometer reading was used as a reference hydration level in the data analysis (Appendix A). The fact that the fractional frequency shift data show greater correlation with the Corneometer measurement also supports that more water is lost on the surface. Large standard deviations are observed in six Corneometer measurements (three separate contacts, each contact in duplication) at each time frame, as indicated by the error bar, while 15 microwave measurements show very small deviation, where the error bars were too small to be visible in the plot. Additionally, the fluctuation of data over time is much smaller in the resonant coaxial probe. On the other hand, the dip seen at time t = 150 min in the microwave measurement shows a drawback of the technique which is due to change in probe-skin contact quality, such as probing location, sample inhomogeneity, or error in contact pressure control.

**Figure 10:**
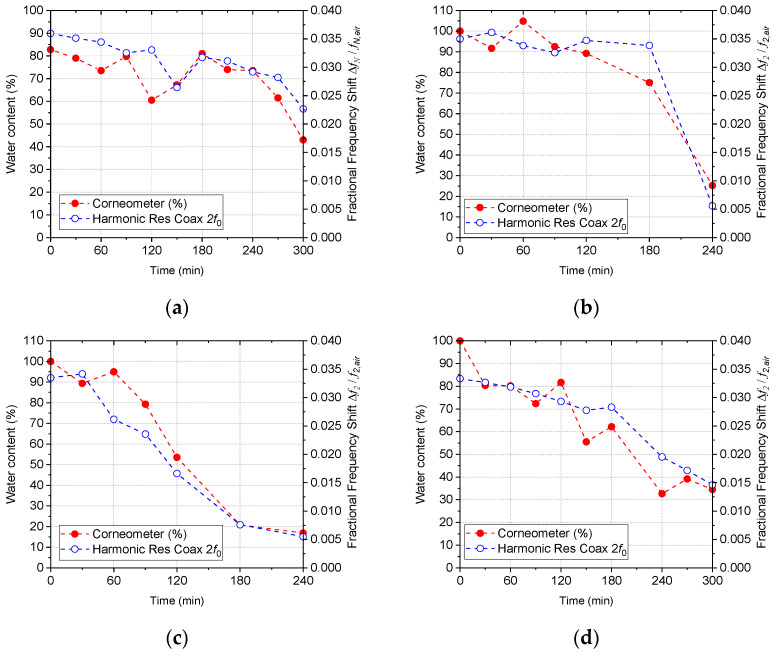
Water content over time measured by Corneometer and fractional frequency shift measured by the proposed harmonic resonance coaxial probe at 2nd harmonic frequency, (**a**) cervical sample A (030215), (**b**) cervical sample B (DT6070), (**c**) cervical sample C (WE4388), and (**d**) cervical sample D (180316).

Comparison of water content of four different cervical samples over time and the fractional frequency shift at the second harmonic frequency is shown in Figure 10. Overall, microwave resonant probe measurement shows good agreement with the Corneometer measurement, following a linear trend of losing water content over time via a natural drying process. Different samples show different rates of change due to sample-to-sample variation. It is clear that the microwave measurement produces a more linear trend than the Corneometer reading, showing less fluctuation over time, except for the sample B (DT6070) in Figure 10b.

**Figure 11:**
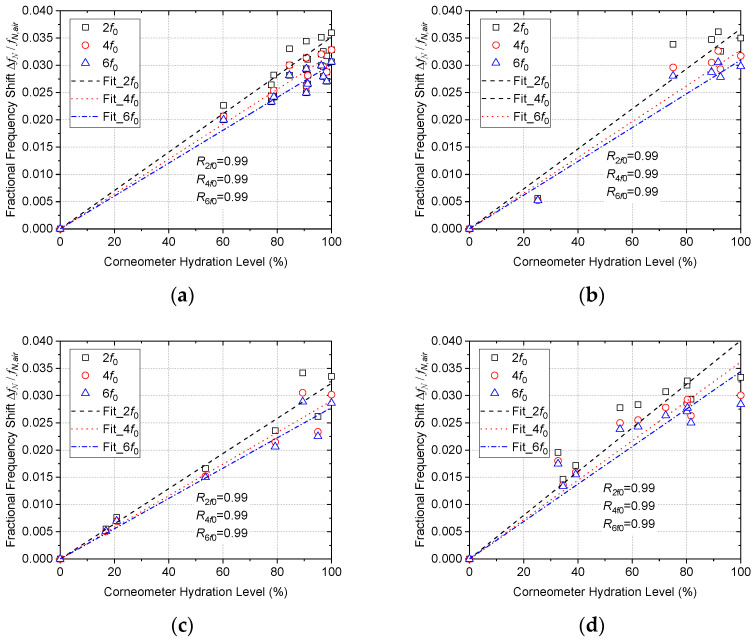
Correlation between Corneometer reading and fractional frequency shift data of the first three even harmonic resonances, (**a**) cervical sample A (030215), (**b**) cervical sample B (DT6070), (**c**) cervical sample C (WE4388), (**d**) cervical sample D (180316).

All three resonant parameters (frequency, 3 dB bandwidth, and peak power) are found to correlate strongly with hydration levels, with frequency chosen here as it gives the highest values of the Pearson’s linear correlation coefficient, *R*. Figure 11 shows the linear correlation between Corneometer measurement and fractional frequency shift. Note the universal, linear behavior exhibited in the plots. This is to be expected, since water content, rather than tissue material, will dominate the microwave response due to its high dielectric constant. It should be noted that when the harmonic resonance coaxial probe is dipped into PBS (phosphate-buffered saline) a fractional frequency shift of 0.038 is obtained for the 350 MHz, decreasing slightly with the mode number. This is perfectly consistent with the cervix hydration data, where an average value of 0.034 ± 0.001 is found. No error bars are plotted here, as it is left to the scatter in the data to indicate the error. It should be noted that the primary source of systematic error is presented by the Corneometer. Considering the nonresonant electrical-impedance-based sensing mechanism of the Corneometer, the microwave resonant sensing technique is expected to be a much more accurate and error-free method of assessing hydration level. On a practical note, measurement of each mode takes less than 2 s, so 10 modes are measured and recorded within 20 s. In a final device, with bespoke and optimized electronics, it is expected that only three modes would need to be measured for reliable hydration levels to be determined, and for the measurement and data recording to be completed within only 2 s.

In terms of sensitivity, the proposed sensor shows mean fractional frequency shift of (3.3 ± 0.3) × 10^−4^ per unit % over the entire data collected (e.g., four different samples and three harmonic resonances). This translates into an absolute frequency shift ($\Delta f_{N})$ of 252 ± 23 kHz/%, 455 ± 41 kHz/%, and 647 ± 57 kHz/% at second, fourth, and sixth harmonic resonance, respectively.

## 5. Conclusions

A microwave resonant open coaxial probe sensor with harmonic resonances was designed for noninvasive human cervical tissue hydration-level monitoring. The estimated hydration level measured by the proposed resonant open coaxial probe shows high and linear correlation compared with the data collected by a commercial skin hydration sensor. This was expected due to the fact that water has a high dielectric constant, and there will be high dielectric contrast as the water content changes in the cervical tissue samples. From a series of in vitro experiments on human cervix tissue samples, we can conclude that the proposed probe has been shown to have high accuracy and good precision thanks to its resonant characteristic with high Q factor.

As discussed in Section 3.2, tight monitoring and control of error sources is the key to obtaining reliable, repeatable, and clinically meaningful data. Further in vivo study would require design modifications and relevant ethical approval process. Finally, we note that a probe of this sort, based on an RG401 coaxial cable and enclosed in a suitable polymer casing, would be a convenient geometry for such noninvasive in vivo testing and form the basis of a medical diagnostic device.

## Figures and Tables

**Table 1:** Measured (air) results of the harmonic resonance coaxial probe.

Harmonic	Frequency	Measured f0	Bandwidth	QL	Q0	Loss
Number	(MHz)	(MHz)	(MHz)			(dB)
1*f*	350	352.6	1.0	337.5	351.1	28.3
2*f*	700	705.3	1.6	448.9	496.0	20.5
3*f*	1050	1058.4	2.2	488.4	569.6	16.9
4*f*	1400	1413.3	2.8	505.1	613.8	15.0
5*f*	1750	1766.0	3.5	503.1	624.0	14.3
6*f*	2100	2116.9	4.2	509.3	625.8	14.6
7*f*	2450	2471.6	5.0	495.1	592.6	15.7
8*f*	2800	2824.5	5.7	495.6	560.6	18.7
9*f*	3150	3182.3	7.5	425.1	451.4	24.7
10*f*	3500	3531.3	N/A	N/A	N/A	32.1
11*f*	3850	3883.7	8.5	455.3	507.9	19.7
12*f*	4200	4237.5	9.5	445.0	552.0	14.3
13*f*	4550	4590.1	11.2	411.4	560.0	11.5
14*f*	4900	4942.9	13.5	376.2	588.0	8.9
15*f*	5250	5297.2	13.3	397.3	679.4	7.6
16*f*	5600	5650.5	15.0	376.3	726.4	6.3
17*f*	5950	6002.8	16.9	355.1	754.2	5.5
18*f*	6300	6355.8	19.9	319.9	699.5	5.3
19*f*	6650	6707.7	23.4	286.3	692.9	4.6
20*f*	7000	7060.8	22.6	312.3	749.6	4.7
21*f*	7350	7413.3	24.7	299.2	732.6	4.6
22*f*	7700	7767.4	25.5	304.3	732.0	4.7
23*f*	8050	8122.5	30.3	268.0	583.4	5.3

**Figure A1:**
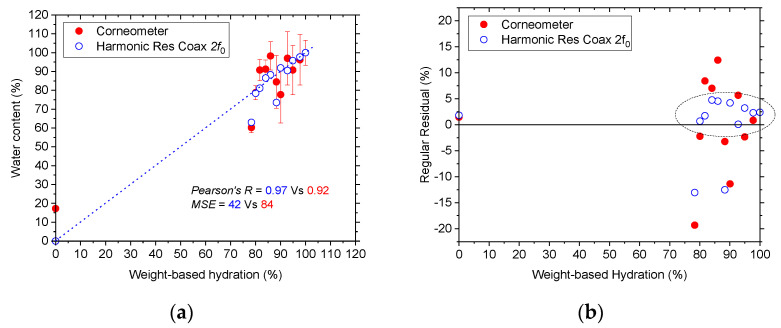
Accuracy comparison of Corneometer and the harmonic resonant coaxial probe with respect to weight-based water content for sample A. (**a**) correlation chart, and (**b**) regular residual data after linear fit.

## Data Availability

Not applicable.

## Appendix A

Fractional frequency shift data can be converted into a relative hydration level (%) either by simply normalizing a selected harmonic resonance to the baseline fractional frequency shift value at time *t* = 0, i.e., when the sample was taken out of suspension liquid, as 100% hydration, or by finding a linear fit equation. Figure A1 shows the comparison of the Corneometer reading and the proposed harmonic resonance coaxial probe measurements for sample A. For fair comparison, all the Corneometer readings were offset by 17.3% so the reading at time *t* = 0 matches 100%. Pearson’s linear correlation coefficient for both measurements seem to be similar, while microwave technique shows a little bit more correlation (0.97 compared with 0.92). However, the mean square error (MSE) is much smaller for the microwave technique, showing half the value of the Corneometer measurements (42 compared with 84), which is also clearly indicated as the grouping in a smaller area in the Regular Residual plot shown in Figure A1b.
